# Psychological distress and lower health-related quality of life are associated with need for dietary support among colorectal cancer survivors with overweight or obesity

**DOI:** 10.1007/s00520-021-06306-6

**Published:** 2021-06-17

**Authors:** Dominique Ramp, Floortje Mols, Nicole Ezendam, Sandra Beijer, Martijn Bours, Renate Winkels, Jolanda de Vries, Jaap C. Seidell, Ellen Kampman, Meeke Hoedjes

**Affiliations:** 1 Center of Research On Psychology in Somatic Diseases, Department of Medical and Clinical Psychology, Tilburg University, Tilburg, the Netherlands; 2 Netherlands Comprehensive Cancer Organisation (IKNL), Utrecht, the Netherlands; 3 Department of Epidemiology, GROW-School for Oncology and Developmental Biology, Maastricht University, Maastricht, the Netherlands; 4 Division of Human Nutrition, Wageningen University, Wageningen, the Netherlands; 5 Department of Health Sciences and the EMGO+ Institute for Health and Care Research, VU University Amsterdam, Amsterdam, the Netherlands

**Keywords:** Colorectal cancer, Dietary support, Health-related quality of life, Obesity, Overweight, Psychological distress

## Abstract

**Objective:**

Two-third of colorectal cancer (CRC) survivors are overweight or obese. Psychological distress and low health-related quality of life (HRQoL) may be barriers to improving diet. We aimed to assess associations between psychological distress and HRQoL and the need for dietary support in CRC survivors with overweight or obesity.

**Methods:**

All alive individuals diagnosed with CRC between 2000 and 2009, as registered by the Dutch population-based Eindhoven Cancer Registry, were eligible for participation and received a questionnaire. Multivariable logistic regression analyses were conducted to assess associations between HRQoL (EORTC QLQ-C30), symptoms of anxiety and depression (HADS), and self-reported need for dietary support (single-item).

**Results:**

A total of 1458 completed the questionnaire (response rate 82%), and 756 (43%) had a BMI of 25.0 or higher and complete data on “need for dietary support” and were included for analyses. BMI ranged between 25.0 and 60.6 (mean, 28.9; SD, 3.6). The majority (71.7%) was overweight (BMI ≥ 25), and 28.3% obese (BMI ≥ 30). Twenty-one percent reported a need for dietary support which was associated with more psychological distress and lower HRQoL. Those who experienced symptoms of anxiety or depression were more likely to report a need for dietary support (27.6% and 28.7%) than those who did not experience symptoms of anxiety (12.3%; OR 2.02; 95% CI 1.22–3.35) or depression (13.5%; OR 1.96; 95% CI 1.19–3.22).

**Conclusions:**

Results suggest that psychological distress and lower HRQoL should be taken into account while promoting a healthy diet in overweight or obese CRC survivors since these factors may hinder adherence to a healthy diet.

## Background

Colorectal cancer (CRC) survivors are recommended to meet lifestyle (i.e., dietary, physical activity [5]) and body weight recommendations to improve both short- and long-term health outcomes [24]. However, research has shown that lifestyle and body weight are suboptimal in CRC survivors [29, 37]. For example, observational studies have shown that about two-thirds of CRC survivors does not meet the recommendation on body fatness [29, 35, 37]. Since body fatness is a well-established independent risk factor for the development of CRC [18, 23], a large proportion of CRC survivors are overweight or obese at the time of diagnosis.

For individuals with overweight or obesity, receiving information or advice is typically not sufficient to be able to improve lifestyle and to maintain these improvements [11]. This implies a need for additional behavioral counseling aimed at self-regulation of lifestyle behavior to be able to adhere to lifestyle advice, rather than provision of lifestyle advice alone. For CRC survivors, receiving such support to be able to eat healthier (i.e., dietary support) is particularly relevant, due to the favorable effects dietary changes may have on frequently reported bowel complaints related to CRC and its treatment, such as diarrhea, increased stool frequency, incontinence, and intolerance of certain foods [9]. Previous research has shown that CRC survivors with overweight and obesity are more likely to report a need for dietary support to be able to eat healthier than those with a body mass index in the normal range [13]. However, dietary support is currently not routinely provided during follow-up care for CRC survivors with overweight and obesity [36].

Dietary support should be offered to CRC survivors with overweight or obesity, as health benefits may be achieved by sustained lifestyle changes in this specific target group in which multiple individual risk factors for morbidity and mortality co-occur. CRC [8], overweight, and obesity [7] have individually been related to an increased risk of diabetes mellitus type 2 [16], cardiovascular diseases [38], (second) primary cancers [20], and/or mortality [38]. A reduction of these risks may be achieved with lifestyle (including dietary) support leading to favorable changes in body weight and diet quality [14].

Dietary support for CRC survivors with overweight or obesity should incorporate support in dealing with CRC-related barriers to achieve and maintain dietary changes [25], including disease and treatment-related complaints [2, 13], such as psychological distress. Psychological distress is common in CRC survivors [19, 22] and in individuals with overweight or obesity [17], and has been associated with an unhealthier lifestyle [6, 31, 32] and a poorer health-related quality of life (HRQoL) in CRC survivors [15, 19]. In addition, HRQoL has been negatively associated with meeting lifestyle and body weight recommendations in CRC survivors [3, 12, 35]. Nevertheless, psychological distress and HRQoL are not commonly taken into account while promoting a healthy lifestyle in CRC survivors with overweight or obesity.

More insight into psychological distress and HRQoL among overweight or obese CRC survivors who report a need for dietary support will inform those providing dietary support in this particular target population about aspects that should be taken into account or incorporated while providing dietary support. Therefore, this study aimed to assess associations between the psychological distress and HRQoL and the need for dietary support in CRC survivors with overweight or obesity. Our hypothesis is that a higher level of psychological distress and a lower HRQoL is associated with a higher need for dietary support in CRC survivors with overweight or obesity.

## Methods

### Study design

To answer our research question, we used cross-sectional data from a larger, population-based prospective observational study, with yearly surveys in Dutch CRC survivors. Details on this longitudinal study can be found elsewhere [4]. The longitudinal study was approved by a Dutch Medical Ethical Committee.

### Study population and setting

All patients diagnosed with CRC from January 2000 to June 2009 were sampled from the southern area of the Netherlands Cancer Registry (NCR). The NCR includes clinical data on all newly diagnosed cancer patients in the Netherlands. Patients with cognitive impairments, unverifiable addresses, and patients who died before the start of the study were excluded.

Patient Reported Outcomes Following Initial Treatment and Long term Evaluation of Survivorship (PROFILES) registry was used for data collection management (https://www.profilesregistry.nl) [33]. Using the PROFILES registry, data on patient-reported outcomes was collected via online or paper questionnaires and linked to clinical data from the NCR.

### Data collection

**Fig. 1:**
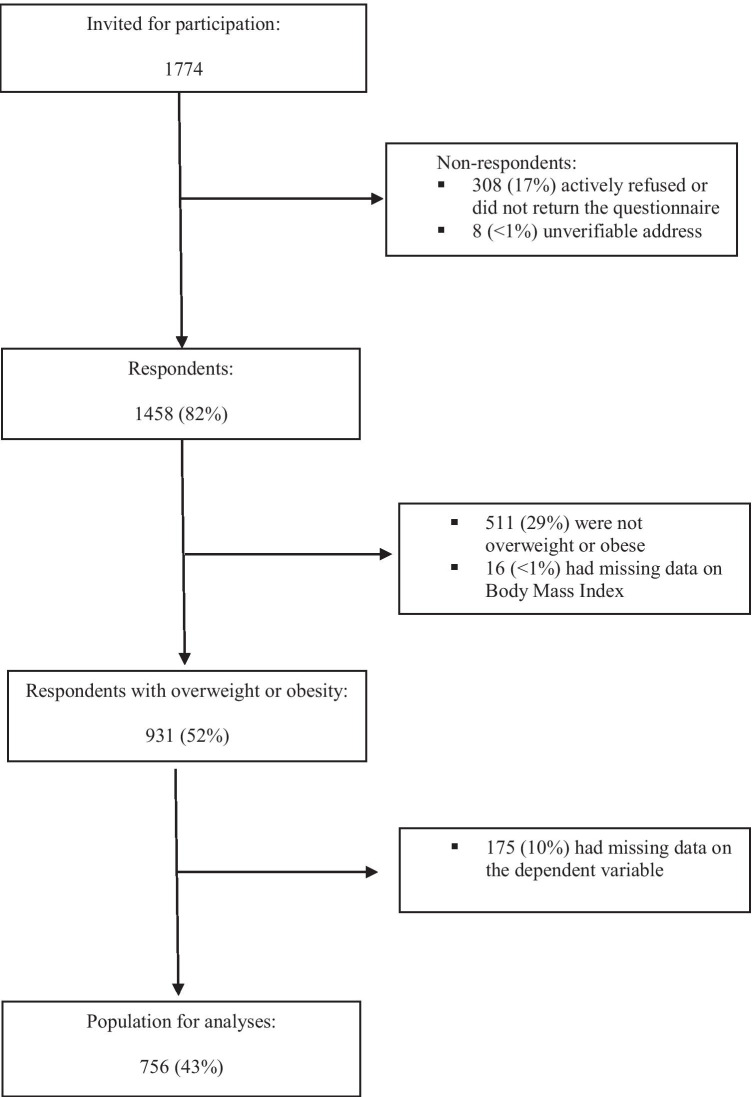
Flowchart of study participants. For this study, cross-sectional data from a larger longitudinal study among colorectal cancer survivors was used. A flowchart of the participants in the longitudinal study has been published elsewhere [4]. This current study involves data obtained from survey 3 in December 2012

The study was set up in December 2010. The present study was performed with data collected in December 2012 as part of the third survey from this longitudinal study, which included an item on the need for dietary support. A total of 1774 eligible CRC patients were then invited for participation via a letter from their (ex-) attending specialist (Fig. 1) and were asked to complete a questionnaire. After 2 weeks, a reminder was sent. All participants provided written informed consent.

### Outcome measures

#### Need for dietary support

A single item was used to assess the need for dietary support. Participants were asked whether they agreed or disagreed with the statement “I feel the need for support to be able to eat healthier.”

#### Sociodemographic, cancer-, and health-related characteristics

Date of birth, sex, and clinical information including date of cancer diagnosis, tumor stage at diagnosis, tumor site, and primary cancer treatment were obtained from the NCR. Socio-economic status (SES) was based on fiscal data on the national economic value of residences and household income aggregated per postal code [34], and categorized into three categories: low, middle, and high. Data on marital status and received follow-up care after treatment at the time of the survey were self-reported using single survey items. “Having diabetes at the moment of the survey or during the 12 months before” (yes/ no) was measured with the adjusted Self-Administered Comorbidity Questionnaire (SCQ) [26]. Having a stoma was assessed by an item from the CRC-specific module of the European Organization for Research and Treatment of Cancer Quality of Life Questionnaire (EORTC QLQ-CR38) [30]. Self-reported body height (cm) and weight (kg) were used to calculate body mass index (BMI). BMI was categorized as underweight (BMI < 18.5 kg/m^2^), normal weight (18.5 ≤ BMI < 25 kg/m^2^), overweight (25 ≤ BMI < 30 kg/m^2^), or obesity (BMI ≥ 30 kg/m^2^).

#### Health-related quality of life

HRQoL was measured using the global quality of life scale and the five functional scales of the European Organization for Research and Treatment of Cancer Quality of Life Questionnaire Core 30 (EORTC QLQ-C30) version 3.0 [1]. The global quality of life scale consists of two items on a 7-point Likert scale, ranging from 1 = “Very bad” to 7 = “Excellent.” The five functional scales consist of items on a 4-point Likert scale, ranging from “Not at all” to “Very much”: physical functioning (5 items), role functioning (2 items), emotional functioning (4 items), cognitive functioning (2 items), and social functioning (2 items). For each scale, the RawScore was composed by calculating the mean of the scale items. According to the scoring manual, scores were transformed to a linear scale ranging from 0 to 100, with higher scores indicating better global quality of life and functioning [10].

#### Psychological distress

Psychological distress was assessed using the Hospital Anxiety and Depression Scale (HADS) [39]. The HADS consists of 14 items assessing self-reported symptoms of anxiety (7 items) and depression (7 items) in the past week. Each item is scored on a 4-point Likert scale, ranging from 0 to 3. A total score can be calculated for both symptoms of anxiety and symptoms of depression by adding up the scores on the 7 items. These total scores range from 0 to 21, with higher scores indicating more symptoms. A cut-off score of 8 or higher is used to indicate having symptoms of depression or anxiety [39].

### Statistical analyses

Statistical analyses were performed using IBM SPSS Statistics for Windows, Version 20. Differences in sociodemographic and clinical characteristics between those included in the population for analyses and those excluded were assessed using independent Student’s t tests for continuous variables and Chi-square tests for categorical variables. Since the variable age at the time of survey completion was not normally distributed, this variable was incorporated in the statistical analyses as a categorical variable. Age was categorized into the following categories based on the observed data distribution: 31–64, 65–69, 70–76, 77–87.

Mean (with standard deviations (SD)) and frequency tables were used to describe sociodemographic, cancer-related, and health-related characteristics. Frequency tables were used to describe psychological distress and cancer-specific HRQoL. Since cancer-specific HRQoL-scores were not normally distributed, scores on global QoL and functional scales were divided into categories for the statistical analyses based on EORTC QLQ-CR30 reference values [28]: “below average” (score between 0 and 66.6), “average” (score between 66.7 and 74.9), “above average” (score between 75.0 and 99.9), and “high” (score of 100).

Univariable logistic regression analyses were conducted, with the need for dietary support (yes/no) as dependent variable and one sociodemographic, cancer-related, or health-related characteristic as independent variable (see Table 1).

**Table 1:** Sociodemographic, cancer-related, and health-related characteristics of colorectal cancer survivors with overweight (N = 756) with and without a need for dietary support

	Total *N* = 756	Need for support *N* = 160	No need for support *N* = 596	
	N (%) *	N (%) *	N (%) *	OR (95%CI)
Age in years				
31–64	216 (28.6)	63 (39.4)	153 (25.7)	1
65–69	158 (20.9)	26 (16.3)	132 (22.1)	0.48 (0.29–0.80)**
70–76	203 (26.9)	43 (26.9)	160 (26.8)	0.65 (0.42–1.02)
77–87	179 (23.7)	28 (17.5)	151 (25.3)	0.45 (0.27–0.74)**
Sex				
Male	507 (67.1)	94 (58.8)	413 (69.3)	0.63 (0.44–0.90)*
Female	249 (32.9)	66 (41.2)	183 (30.7)	1
Marital status				
Living without a partner	150 (19.8)	44 (27.8)	106 (17.8)	1.78 (1.19–2.67)**
Living with a partner	603 (79.8)	114 (72.2)	489 (82.2)	1
SES				
Low	130 (17.2)	37 (23.9)	93 (16.5)	1.62 (1.00–2.61)
Medium	300 (39.7)	61 (39.4)	239 (42.2)	1.04 (0.69–1.56)
High	289 (38.2)	57 (36.8)	232 (41.1)	1
Tumor site				
Colon	430 (56.9)	87 (54.4)	343 (57.6)	0.88 (0.62–1.25)
Rectum	326 (43.1)	73 (45.6)	253 (42.4)	1
Tumor stage at diagnosis				
Stage I	220 (29.1)	46 (29.9)	174 (30.0)	1
Stage II	250 (33.1)	53 (34.4)	197 (34.0)	1.02 (0.65–1.59)
Stage III	244 (32.3)	52 (33.8)	192 (33.1)	1.02 (0.66–1.60)
Stage IV	20 (2.6)	3 (1.9)	17 (2.9)	0.67 (0.19–2.38)
Time since diagnoses in years				
(Mean (SD))	6.8 (2.7)	6.8 (2.6)	6.7 (2.8)	1.02 (0.95–1.08)
Receiving follow-up care				
No	188 (24.9)	39 (24.8)	149 (25.2)	1
Yes	560 (74.1)	118 (75.2)	442 (74.8)	1.02 (0.68–1.53)
Treatment				
Surgery only	325 (43.0)	67 (41.9)	258 (43.4)	**
Surgery + radio	178 (23.5)	39 (24.4)	139 (23.4)	
Surgery + chemo	172 (22.8)	38 (23.8)	134 (22.5)	
Surgery + radio + chemo	78 (10.3)	15 (9.4)	63 (10.6)	
Chemotherapy only	2 (0.3)	1 (0.6)	1 (0.2)	
Chemotherapy				
No	504 (66.7)	106 (66.3)	398 (66.8)	1
Yes	252 (33.3)	54 (33.8)	198 (33.2)	1.02 (0.71–1.48)
Radiotherapy				
No	500 (66.1)	106 (66.3)	394 (66.1)	1
Yes	256 (33.9)	54 (33.8)	202 (33.9)	0.99 (0.69–1.44)
Stoma				
No	503 (66.5)	98 (70.5)	405 (76.3)	1
Yes	167 (22.1)	41 (29.5)	126 (23.7)	1.35 (0.89–2.04)
Diabetes				
No	526 (69.6)	94 (71.2)	432 (83.1)	1
Yes	126 (16.7)	38 (28.8)	88 (16.9)	1.99 (1.28–3.09)**
Degree of overweight				
Overweight	542 (71.7)	95 (59.4)	447 (75.0)	1
Obese	214 (28.3)	65 (40.6)	149 (25.0)	2.05 (1.42–2.96)***

Statistically significantly associated variables are printed bold

*Abbreviations*: *SD*, standard deviation; *SES*, socio-economic status; *BMI*, body mass index; *OR*, odds ratio; *CI*, confidence interval

^*^Percentages do not always add up to 100% because of missing values

^**^Chi-square cannot be calculated since 2 cells (20%) have an expected count of less than 5. * p < .05, ** p < .01, *** p < .001

Logistic regression analyses were conducted to examine associations between HRQoL, psychological distress, and the need for dietary support. Univariable logistic regression analyses were conducted with need for support as dependent variable and a single HRQOL variable or psychological distress variable as independent variable. Multivariable logistic regression analyses were conducted with need for support as dependent variable, and as independent variables one cancer-specific HRQoL variable or psychological distress variable and all sociodemographic, cancer-related, and health-related characteristics that were found to be statistically significantly associated with the need for support in the univariable logistic regression analyses (age, sex, having a partner, having diabetes, degree of overweight) (see Table 2).

**Table 2:** Associations between need for dietary support, psychological distress, and cancer-specific health-related quality of life among colorectal cancer survivors with overweight/obesity

	Total *N* = 756	Need for support *N* = 160	No need for support *N* = 596	Univariable^a^	Multivariable^b^
	N (%)	N (%)	N (%)	OR (95%CI)	OR (95%CI)
Psychological distress					
Anxiety					
Yes^c^	115 (15.5)	43 (27.6)	72 (12.3)	2.71 (1.77–4.16)***	2.02 (1.22–3.35)**
No	626 (84.5)	113 (72.4)	513 (87.7)	1	1
Depression					
Yes^c^	124 (16.7)	45 (28.7)	79 (13.5)	2.58 (1.70–3.92)***	1.96 (1.19–3.22)**
No	619 (83.3)	112 (71.3)	507 (86.5)	1	1
Cancer-specific HRQoL					
Global quality of life					
Below average^d^	113 (15.1)	42 (26.8)	71 (12.1)	1	1
Average	113 (15.1)	29 (18.5)	84 (14.3)	0.58 (0.33–1.03)	0.83 0(.42–1.61)
Above average	405 (54.3)	68 (43.3)	337 (57.2)	0.34 (0.22–0.54)***	0.50 (0.29–0.86)*
High	115 (15.4)	18 (11.5)	97 (16.5)	0.31 (0.17–0.59)***	0.42 (0.20–0.88)*
Physical functioning					
Below average^d^	125 (18.0)	45 (28.5)	90 (15.2)	1	1
Average	100 (13.4)	25 (15.8)	75 (12.7)	0.67 (0.37–1.19)	0.62 (0.31–1.24)
Above average	328 (43.8)	61 (38.6)	267 (45.2)	0.46 (0.29–0.72)**	0.54 (0.31–0.94)*
High	86 (24.8)	27 (17.1)	159 (26.9)	0.34 (0.20–0.59)***	0.39 (0.20–0.76)**
Role functioning					
Below average^d^	125 (16.7)	40 (25.3)	85 (14.4)	1	1
Average	130 (17.4)	36 (22.8)	94 (15.9)	0.81 (0.48–1.39)	0.99 (0.53–1.85)
Above average	74 (9.9)	19 (12.0)	55 (9.3)	0.73 (0.39–1.40)	1.13 (0.53–2.37)
High	419 (56.0)	62 (39.9)	356 (60.3)	0.38 (0.24–0.60)***	0.51 (0.29–0.88)*
Emotional functioning					
Below average^d^	70 (9.4)	31 (19.6)	39 (6.6)	1	1
Average	62 (8.3)	12 (7.6)	50 (8.5)	0.30 (0.14–0.66)**	0.32(0.12–0.80)*
Above average	219 (29.3)	45 (28.5)	174 (29.5)	0.33 (0.18–0.58)***	0.43(0.22–0.84)*
High	397(53.1)	70 (44.3)	327 (55.4)	0.27 (0.16–0.46)***	0.41(0.22–0.78)**
Cognitive functioning					
Below average^d^	71 (9.5)	25 (15.8)	46 (7.8)	1	1
Average	107 (14.3)	30 (19.0)	77 (13.1)	0.72 (0.38–1.37)	0.73(0.34–1.59)
Above average	210 (28.1)	51 (32.3)	159 (26.9)	0.59 (0.33–1.05)	0.89(0.45–1.76)
High	360 (48.1)	52 (32.9)	308 (52.2)	0.31 (0.18–0.55)***	0.39(0.20–0.77)**
Social functioning					
Below average^d^	61 (8.2)	24 (15.4)	37 (6.3)	1	1
Average	110 (14.7)	33 (21.2)	77 (13.1)	0.66 (0.34–1.27)	0.65(0.30–1.37)
Above average	104 (13.9)	20 (12.8)	84 (14.2)	0.37 (0.18–0.75)**	0.39(0.17–0.88)*
High	471 (63.1)	79 (50.6)	392 (66.4)	0.31 (0.18–0.55)***	0.37(0.19–0.70)**

Abbreviations: *HRQoL*, health-related quality of life; *SD*, standard deviation; *OR*, odds ratio; *CI*, confidence interval

^*^ p < .05

^**^ p < .01

^***^ p < .001

^a^Univariable logistic regression analyses with need for support as dependent variable and a single cancer-specific health-related quality of life variable or psychological distress variable as independent variable

^b^Multivariable logistic regression analyses with need for support as dependent variable, and independent variables: age, gender (male/female), having a partner (yes/no), having diabetes (yes/no), degree of overweight (overweight/obese), and one cancer-specific health-related quality of life variable or psychological distress variable

^c^A cut-off score of 8 or higher on anxiety or depression on the Hospital Anxiety and Depression Scale (HADS) is defined as having symptoms of anxiety (yes/no) or depression (yes/no)

^d^ “Below average” represents a score between 0 and 66.6, “average” represents a score between 66.7 and 74.9, “above average” represents a score between 75.0 and 99.9, and “high” represents a score of 100 on the European Organization for Research and Treatment of Cancer Quality of Life Questionnaire Core 30 (EORTC QLQ-CR30)

## Results

Of the 1774 CRC survivors who were invited for participation, 1458 completed the questionnaire (response rate 82%), and 756 (43%) had a BMI of 25.0 or higher and complete data on “need for dietary support” and were included for analyses (Fig. 1). Compared to those included for analyses, those who were excluded were older (mean age 70.9, SD 9.5 vs. mean age 68.8, SD 9.4; *p* < 0.001), more often female (50.6 vs. 32.9%; *p* < 0.001), more often diagnosed with colon cancer (62.7 vs. 56.9%; *p* = 0.014), more often diagnosed at an earlier tumor stage (stage I 31.3%, stage II 38.5% vs. stage I 30.0% and stage II 34.1%; *p* = 0.038), and less often underwent chemotherapy (27.6 vs. 33.3%, *p* = 0.009). Those excluded did not differ from those included with regard to socio-economic status and time since diagnosis.

Age of CRC survivors in the population for analyses ranged from 35 to 87 years, with a mean age of 68.8 (SD 9.4) (Table 1). Most were male (67.1%) and living with a partner (79.8%). Time since diagnosis varied from 3 to 13 years (mean 6.8 years; SD 2.7). BMI ranged between 25.0 and 60.6 (mean 28.9; SD 3.6). The majority (71.7%) was classified as overweight (BMI ≥ 25) and 28.3% as obese (BMI ≥ 30).

### Need for dietary support

A need for dietary support was reported by 21.2% (n = 160). Compared with those without a need for dietary support, CRC survivors who reported a need for dietary support were younger (mean age 66.5, SD = 10.6 vs. mean age 69.4, SD = 8.9), more often female (41.2 vs. 30.7%), less often had a partner (72.2 vs. 82.2%), more often had diabetes (28.8 vs. 16.9%), and were more often classified as obese (40.6 vs. 25.0%) (all *p* < 0.05; Table 1).

### Health-related quality of life

All HRQoL domains were significantly associated with the need for dietary support. Univariable and multivariable logistic regression analyses showed comparable results and are discussed below (Table 2). A total of 69.7% of overweight and obese CRC survivors had either high (15.4%) or above average (54.3%) global QoL scores. Similarly, the majority of them had either high or above average scores across functional scales. The largest majorities of high and above average scores were observed in the emotional functioning (82.4%), the social functioning (77.0%), and the cognitive functioning (76.2%) scales.

#### Global quality of life

A total of 15.1% had a global QoL score below average. CRC survivors who reported a need for dietary support more often had a global QoL score below average (26.8%) than CRC survivors without a need for dietary support (12.1%). Compared with those with a global QoL score below average, those with a high global QoL score and those with a global QoL score above average were less likely to report a need for dietary support (high OR 0.42; 95% CI 0.20–0.88; above average OR 0.50; 95% CI 0.29–0.86; Table 2).

#### Functional scales

Among the functional scales, the largest proportions of below average scores were observed in the physical functioning (18.0%) and the role functioning (16.7%) scales. All functional scales were statistically significantly associated with the need for dietary support (Table 2).

CRC survivors who reported a need for dietary support more often had a physical functioning score below average (28.5%) than CRC survivors without a need for dietary support (15.2%). Compared with those with a physical functioning score below average, those with a high physical functioning score and those with a physical functioning score above average were less likely to report a need for dietary support (high OR 0.39; 95% CI 0.20–0.76; above average OR 0.54; 95% CI 0.31–0.94).

CRC survivors who reported a need for dietary support more often had a role functioning score below average (25.3%) than CRC survivors without a need for dietary support (14.4%). Compared with those with a role functioning score below average, those with a high role functioning score were less likely to report a need for dietary support (OR 0.51; 95% CI 0.29–0.88).

### Psychological distress

Psychological distress was significantly associated with the need for dietary support. Univariable and multivariable logistic regression analyses showed comparable results (Table 2). Symptoms of anxiety were reported by 15.5% of CRC survivors. Those who experienced symptoms of anxiety were more likely to report a need for dietary support (27.6%) than those who did not experience symptoms of anxiety (12.3%; OR 2.02; 95% CI 1.22–3.35).

Symptoms of depression were reported by 16.7%. Participants with symptoms of depression were more likely to report a need for dietary support (28.7%) compared with those without symptoms of depression (13.5%; OR 1.96; 95% CI 1.19–3.22).

## Discussion

To the best of our knowledge, this is the first study on the associations of psychological distress and HRQoL with the need for dietary support among CRC survivors with overweight or obesity. We observed that those with symptoms of anxiety and depression and a lower HRQoL (all domains) were more likely to report a need for dietary support compared to those without a need for dietary support.

Previous research on psychological health and lifestyle behavior among CRC survivors has shown an association between higher psychological distress and an unhealthier lifestyle [6, 31, 32]. Trudel-Fitzgerald et al. [31] found that among women with CRC from the Nurses’ Health Study prospective cohort in the USA, higher levels of anxiety and depression symptoms were associated with subsequent unhealthier lifestyle in the 10 years following CRC diagnosis [31]. Although, to our knowledge, no other study has specifically assessed the association between psychological distress and diet quality among CRC survivors, two studies have assessed the association between psychological distress and physical (in)activity in CRC survivors [6, 32]. In a prospective survey among Australian CRC survivors, it was found that those who experienced anxiety were at greater risk of physical inactivity. In addition, CRC survivors with higher levels of initial anxiety were less likely to increase their levels of self-reported physical activity at subsequent time points, whereas depression was not found to be related to increases in physical activity [6]. Similarly, a population-based cross-sectional study in Canadian and Western Australian colon cancer survivors showed that higher levels of accelerometer-assessed moderate-to-vigorous intensity physical activity were associated with lower levels of anxiety, but not with symptoms of depression [32].

Previous studies on the association between HRQoL and lifestyle in CRC survivors found that a higher HRQOL was associated with favorable lifestyle or meeting lifestyle recommendations [3, 12, 27, 35], and suggest a positive association between a higher HRQoL and the number of lifestyle recommendations that are being met. Associations seem to be strongest between physical activity and physical HRQoL domain scores (e.g., physical functioning) [27]. Few studies have specifically examined the association between HRQoL and adherence to dietary recommendations or diet quality among CRC survivors [7, 9, 18, 27]. Whereas Grimmett et al. [12] found that a higher global QoL and physical, role, and cognitive function was associated with fruit and vegetable intake in CRC survivors [9, 35, 36] found that HRQoL and HRQoL domains were not associated with adherence to the dietary recommendations shortly after diagnosis [7]. Both studies did not assess the need for dietary support.

### Limitations and strengths

Several limitations need to be taken into account while interpreting our findings. First, the underrepresentation of stage IV CRC survivors observed in our study sample limits the generalizability of our findings to CRC survivors diagnosed at stage IV. Second, due to a lack of power, we were unable to include both psychological distress variables and HRQoL domains in the multivariable logistic regression analyses [21], while ideally we would have included these variables since previous research has shown an association between psychological distress and HRQoL in CRC survivors [15, 19]. Third, the need for dietary support was assessed with a single item which could have been less clear for patients and resulted in less nuanced results compared to the assessment of needs with a scale or questionnaire. Also, one should remember that the expression of a need for dietary support does not automatically mean poor adherence to dietary guidelines. It should be noted that our findings may have possibly been influenced by selection bias. Those included in our study sample were less often female compared with those excluded from our study sample while our findings show that females were more likely to report a need for dietary support. Thus, there may have been an underestimation of the proportion of those in need for dietary support in our study sample. On the other hand, the proportion of those in need for support may have been overestimated since those included in our study sample were younger compared with those excluded while our findings show that being younger was associated with a need for dietary support.

Also, the cross-sectional nature of this study limits the determination of causal associations. Finally, although we did not observe an association between time since diagnosis and the perceived need for dietary support to be able to eat healthier in our study among survivors varying in time since diagnosis (range 3 to 13 years), the need for dietary support most likely will vary over time for the individual CRC survivor, as will perceived levels of psychological distress. Future longitudinal observational studies could provide valuable information on fluctuations over time and individual differences in the need for support, psychological distress, and HRQoL which can inform decisions on when to provide what type of dietary and/or psychological support.

Strengths of this study that are worth mentioning include the relatively large population-based study sample with a high response rate, obtained via the well-established PROFILES registry [33], and the use of standardized, validated, commonly used questionnaires to assess psychological distress and HRQoL [1, 39].

### Implications

Our finding that higher psychological distress and a lower HRQoL were associated with the need for dietary support suggests that these factors, which may hinder adherence to a healthy diet, should be taken into account while promoting a healthy diet in overweight or obese CRC survivors. Ideally, multidisciplinary support should be offered to CRC survivors with overweight and obesity, using a more holistic approach than currently is applied in clinical oncological care, to promote a healthy lifestyle and physical and psychological health.

## Conclusion

This study showed that psychological distress and a lower HRQoL across all domains were associated with the need for dietary support in CRC survivors with overweight or obesity. Results suggest that perceived psychological and physical health should be taken into account while promoting a healthy diet in overweight or obese CRC survivors since these factors may hinder adherence to a healthy diet.

## Data Availability

Data from the PROFILES registry are freely available for non-commercial scientific research, subject to submission of a study question, privacy and confidentiality restrictions, and registration (www.profilesregistry.nl).

## References

[1] Aaronson NK, Ahmedzai S, Bergman B (1993). The European Organization for Research and Treatment of Cancer QLQ-C30: a quality-of-life instrument for use in international clinical trials in oncology. J Natl Cancer Inst.

[2] Anderson AS, Steele R, Coyle J (2013). Lifestyle issues for colorectal cancer survivors–perceived needs, beliefs and opportunities. Support Care Cancer.

[3] Blanchard CM, Courneya KS, Stein K (2008). Cancer survivors’ adherence to lifestyle behavior recommendations and associations with health-related quality of life: results from the American Cancer Society’s SCS-II. J Clin Oncol.

[4] Bours MJ, Beijer S, Winkels RM, van Duijnhoven FJ, Mols F, Breedveld-Peters JJ et al (2015) Dietary changes and dietary supplement use, and underlying motives for these habits reported by colorectal cancer survivors of the Patient Reported Outcomes Following Initial Treatment and Long-Term Evaluation of Survivorship (PROFILES) registry. Br J Nutr 114(2):286–296 10.1017/S0007114515001798 26079602

[5] Buffart LM, Galvao DA, Brug J (2014). Evidence-based physical activity guidelines for cancer survivors: current guidelines, knowledge gaps and future research directions. Cancer Treat Rev.

[6] Chambers SK, Lynch BM, Aitken J (2009). Relationship over time between psychological distress and physical activity in colorectal cancer survivors. J Clin Oncol.

[7] Collaborators GBDO, Afshin A, Forouzanfar MH (2017). Health effects of overweight and obesity in 195 countries over 25 years. N Engl J Med.

[8] De Marco MF, Janssen-Heijnen ML, van der Heijden LH (2000). Comorbidity and colorectal cancer according to subsite and stage: a population-based study. Eur J Cancer.

[9] Denlinger CS, Barsevick AM (2009) The challenges of colorectal cancer survivorship. J Natl Compr Canc Netw 7(8): 883–893; quiz 894 10.6004/jnccn.2009.0058 PMC3110673 19755048

[10] Fayers PM, Aaronson NK, Bjordal K et al (2001) The EORTC QLQC30 Scoring Manual. Reportno. Report Number|, Date. Place Published|: Institution|

[11] Franz MJ, VanWormer JJ, Crain AL (2007). Weight-loss outcomes: a systematic review and meta-analysis of weight-loss clinical trials with a minimum 1-year follow-up. J Am Diet Assoc.

[12] Grimmett C, Bridgewater J, Steptoe A (2011). Lifestyle and quality of life in colorectal cancer survivors. Qual Life Res.

[13] Hoedjes M, de Kruif A, Mols F (2017). An exploration of needs and preferences for dietary support in colorectal cancer survivors: a mixed-methods study. PLoS ONE.

[14] Hoedjes M, van Stralen MM, Joe STA (2017). Toward the optimal strategy for sustained weight loss in overweight cancer survivors: a systematic review of the literature. J Cancer Surviv.

[15] Jansen L, Herrmann A, Stegmaier C (2011). Health-related quality of life during the 10 years after diagnosis of colorectal cancer: a population-based study. J Clin Oncol.

[16] Khaledi M, Haghighatdoost F, Feizi A et al (2019) The prevalence of comorbid depression in patients with type 2 diabetes: an updated systematic review and meta-analysis on huge number of observational studies. Acta Diabetol. 10.1007/s00592-019-01295-9 10.1007/s00592-019-01295-9 30903433

[17] Mannan M, Mamun A, Doi S (2016). Is there a bi-directional relationship between depression and obesity among adult men and women? Systematic review and bias-adjusted meta analysis. Asian J Psychiatr.

[18] McMichael AJ (2008). Food, nutrition, physical activity and cancer prevention. Authoritative report from World Cancer Research Fund provides global update. Public Health Nutr.

[19] Mols F, Schoormans D, de Hingh I (2018). Symptoms of anxiety and depression among colorectal cancer survivors from the population-based, longitudinal PROFILES Registry: Prevalence, predictors, and impact on quality of life. Cancer.

[20] Ng AK, Travis LB (2008) Second primary cancers: an overview. Hematol Oncol Clin North Am 22(2): 271–289, vii 10.1016/j.hoc.2008.01.007 18395150

[21] Peduzzi P, Concato J, Kemper E (1996). A simulation study of the number of events per variable in logistic regression analysis. J Clin Epidemiol.

[22] Peng YN, Huang ML, Kao CH (2019) Prevalence of depression and anxiety in colorectal cancer patients: a literature review. Int J Environ Res Public Health 16(3) 10.3390/ijerph16030411 PMC6388369 30709020

[23] Renehan AG, Tyson M, Egger M (2008). Body-mass index and incidence of cancer: a systematic review and meta-analysis of prospective observational studies. Lancet.

[24] Research WCRFAIfC (2018) Diet, nutrition, physical activity and cancer: a global perspective. Reportno. Report Number|, Date. Place Published|: Institution|

[25] Rock CL, Doyle C, Demark-Wahnefried W et al (2012) Nutrition and physical activity guidelines for cancer survivors. CA Cancer J Clin. 10.3322/caac.21142 10.3322/caac.21142 22539238

[26] Sangha O, Stucki G, Liang MH (2003). The Self-Administered Comorbidity Questionnaire: a new method to assess comorbidity for clinical and health services research. Arthritis Rheum.

[27] Schlesinger S, Walter J, Hampe J (2014). Lifestyle factors and health-related quality of life in colorectal cancer survivors. Cancer Causes Control.

[28] Scott NW, Fayers PM, Aaronson NK et al (2008) EORTC QLQ-C30 reference values. Reportno. Report Number|, Date. Place Published|: Institution|

[29] Soerjomataram I, Thong MS, Korfage IJ et al (2012) Excess weight among colorectal cancer survivors: target for intervention. J Gastroenterol. 10.1007/s00535-012-0567-2 10.1007/s00535-012-0567-2 PMC3443337 22426635

[30] Sprangers MA, te Velde A, Aaronson NK (1999). The construction and testing of the EORTC colorectal cancer-specific quality of life questionnaire module (QLQ-CR38). European Organization for Research and Treatment of Cancer Study Group on Quality of Life. Eur J Cancer.

[31] Trudel-Fitzgerald C, Tworoger SS, Poole EM (2018). Psychological symptoms and subsequent healthy lifestyle after a colorectal cancer diagnosis. Health Psychol.

[32] Vallance JK, Boyle T, Courneya KS (2015). Accelerometer-assessed physical activity and sedentary time among colon cancer survivors: associations with psychological health outcomes. J Cancer Surviv.

[33] van de Poll-Franse LV, Horevoorts N, van Eenbergen M (2011). The Patient Reported Outcomes Following Initial treatment and Long term Evaluation of Survivorship registry: scope, rationale and design of an infrastructure for the study of physical and psychosocial outcomes in cancer survivorship cohorts. Eur J Cancer.

[34] van Duijn C, Keij I (2002). Sociaal-economische status indicator op postcode niveau (Socioeconomic status indicator on zip code level). Maandstatistiek van de bevolking.

[35] van Veen MR, Mols F, Bours MJL et al (2019a) Adherence to the World Cancer Research Fund/American Institute for Cancer Research recommendations for cancer prevention is associated with better health-related quality of life among long-term colorectal cancer survivors: results of the PROFILES registry. Support Care Cancer. 10.1007/s00520-019-04735-y 10.1007/s00520-019-04735-y PMC6825038 30927111

[36] van Veen MR, Mols F, Smeets L et al (2019b) Colorectal cancer survivors’ beliefs on nutrition and cancer; correlates with nutritional information provision. Support Care Cancer. 10.1007/s00520-019-04934-7. 10.1007/s00520-019-04934-7 PMC6989414 31227989

[37] Winkels RM, van Lee L, Beijer S et al (2016) Adherence to the World Cancer Research Fund/American Institute for Cancer Research lifestyle recommendations in colorectal cancer survivors: results of the PROFILES registry. Cancer Med. 10.1002/cam4.791 10.1002/cam4.791 PMC5055169 27418442

[38] Mozaffarian D, Benjamin EJ, Writing Group M (2016). Heart disease and stroke statistics-2016 update: a report from the American Heart Association. Circulation.

[39] Zigmond AS, Snaith RP (1983). The hospital anxiety and depression scale. Acta Psychiatr Scand.

